# *Plasmodium berghei Δp52&p36* Parasites Develop Independent of a Parasitophorous Vacuole Membrane in Huh-7 Liver Cells

**DOI:** 10.1371/journal.pone.0050772

**Published:** 2012-12-05

**Authors:** Ivo H. J. Ploemen, Huib J. Croes, Geert-Jan J. van Gemert, Mietske Wijers-Rouw, Cornelus C. Hermsen, Robert W. Sauerwein

**Affiliations:** 1 Department of Medical Microbiology, Radboud University Nijmegen Medical Center, Nijmegen, The Netherlands; 2 Department of Cell Biology, Radboud University Nijmegen Medical Center, Nijmegen, The Netherlands; Seattle Biomedical Research Institute, University of Washington, United States of America

## Abstract

The proteins P52 and P36 are expressed in the sporozoite stage of the murine malaria parasite *Plasmodium berghei*. *Δp52&p36* sporozoites lacking expression of both proteins are severely compromised in their capability to develop into liver stage parasites and abort development soon after invasion; presumably due to the absence of a parasitophorous vacuole membrane (PVM). However, a small proportion of *P. berghei Δp52&p36* parasites is capable to fully mature in hepatocytes causing breakthrough blood stage infections. We have studied the maturation of replicating *Δp52&p36* parasites in cultured Huh-7 hepatocytes. Approximately 50% of *Δp52&p36* parasites developed inside the nucleus of the hepatocyte but did not complete maturation and failed to produce merosomes. In contrast cytosolic *Δp52&p36* parasites were able to fully mature and produced infectious merozoites. These *Δp52&p36* parasites developed into mature schizonts in the absence of an apparent parasitophorous vacuole membrane as shown by immunofluorescence and electron microscopy. Merozoites derived from these maturing *Δp52&p36* liver stages were infectious for C57BL/6 mice.

## Introduction

*Plasmodium* sporozoites are transmitted to the mammalian host by the bites of infected *Anopheline* mosquitoes. The parasites leave the injection site and make their way to the liver where they invade hepatocytes before commencing the erythrocytic cycle. There are two distinct pathways by which *Plasmodium* sporozoites enter hepatocytes: they either migrate through cells disrupting the host cell membrane, or they invaginate the host cell membrane forming a parasitophorous vacuole (PV) and a parasitophorous vacuole membrane (PVM) [1]. Proper formation and subsequent modification of the PV and PVM are considered crucial for development and survival of intrahepatic parasites [2]. Nonetheless, a small proportion of *Plasmodium* parasites is capable of (partial) intranuclear development [3], [4] in the absence of a PVM [4]. After invasion of the hepatocyte the sporozoites multiply and form tens of thousands of merozoites, which are released into the bloodstream as merosomes.

Both the *Plasmodium* genes *p52*, encoding a putative GPI-anchored protein [5], [6] and its paralogous gene *p36*, encoding a putative secreted protein [6] are upregulated in sporozoite stages [7] with a putative function in hepatocyte invasion. *P. berghei* and *P. yoelii* parasites, genetically attenuated by the deletion of the *p52* gene or the *p36* gene, lack a PVM upon hepatocyte invasion [5], [8]. These mutant parasites are severely compromised in their capability to develop into liver cells and abort development soon after invasion. The developmental arrest of these *Δp*52&p36 mutant parasites was confirmed in *P. falciparum* [7]. Infection of mice with high numbers of *P. yoelii Δp*52&p36 sporozoites, does not result in a blood stage infection [8]. The developmental arrest of these knock-out parasites is thought to be related to the lack of a PVM, considered critical for intracellular survival in hepatocytes.

Despite the apparent full developmental arrest, we previously showed that a low percentage of *Δp52* [5] and *Δp*52&p36 [9] parasites are able to generate a blood stage infection in the *P. berghei* murine model. Moreover, we provided evidence that low numbers of *Δp*52&p36* P. falciparum* sporozoites, develop into replicating liver stages [9].

In this study we followed replicating *Δp*52&p36 parasites in the course of hepatic maturation and more specifically in relation to intranuclear location and PVM development.

## Materials and Methods

### Mice and Parasites

Female C57BL/6J, eight weeks of age, were purchased from Elevage Janvier (France). All studies in which animals were involved have been performed according to the regulations of the Dutch “Animal On Experimentation act” and the European Directive 2010/63/EU.

The *P. berghei Δp*52&p36 and wildtype (*P. berghei* ANKA) parasites used are described elsewhere [9].

### Analysis of in vitro P. berghei Liver-stage Development by Immunofluorescence

*P. berghei* sporozoites were collected at day 21 after mosquito infection by hand-dissection of salivary glands. Salivary glands were collected in DMEM (Dulbecco’s Modified Eagle Medium from GIBCO) and homogenized in a homemade glass grinder. The number of sporozoites was determined by counting samples in duplicate in a Bürker-Türk counting chamber using phase-contrast microscopy.

**Figure 1:**
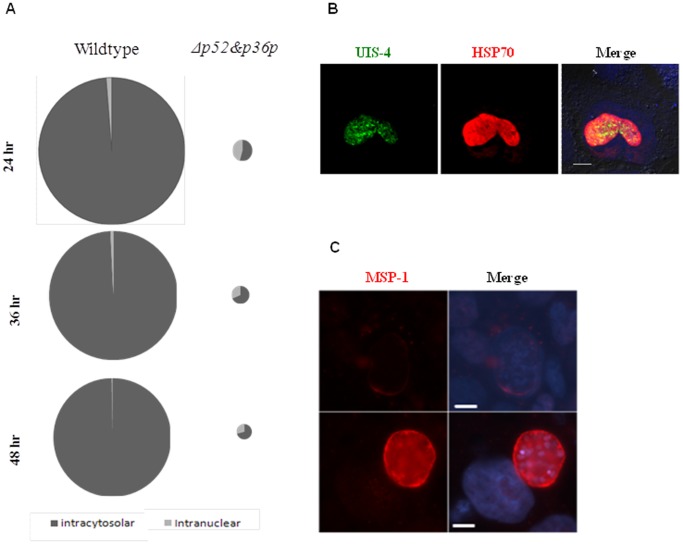
Intranuclear development of *Δp52&p36 P. berghei* parasites. A) Pie diagrams of intranuclear and cytosolic wildtype and mutant replicating parasites at 24, 36 and 48 hours post invasion in Huh-7 cells. The diameter of the circles represents the relative number of replicating parasites observed per coverslip, where the wildtype circle at 24 hour represent 100% and all other circles are deduced (wildtype  = 1300–1500 and Δp52&p36 = 20–40 replicating parasites per coverslip at 24 hours post infection. Absolute numbers are depicted in Table S1 B) UIS-4 and HSP70 expression on an intranuclear *P. berghei* parasite 44 hours post infection (Bar  = 10 µm). C) MSP-1 expression on intranuclear (*Δp52&p36)* and cytocolic (wildtype) *P. berghei* parasites 52 hours post infection (Bar  = 10 µm).

Liver stage development of the *P. berghei* mutants and wildtype parasites was determined *in vitro* as described previously [9]. Briefly, human liver hepatoma cells (Huh-7 [10]) were suspended in 1 ml of ‘complete’ DMEM (DMEM, Gibco, supplemented with 10% FCS, 1% penicillin/streptomycin and 1% Glutamax) and were seeded on coverslips in 24-well plates (10^5^ cells/well). After Huh-7 monolayers were >80% confluent, 5×10^4^ sporozoites were added per well, and centrifuged 10 minutes at 1800×G (eppendorf centrifuge 5810 R). At different time points after infection, cells were fixed with 4% paraformaldehyde, permeabilized with 0.1% Triton-X-100, blocked with 10% FCS in PBS, and subsequently stained with a primary and secondary antibody at room temperature for 45 and 30 min respectively. Primary antibodies used were anti-*Pb*UIS-4 (raised in rabbit; [11], detecting a PVM-resident protein); anti-*Pb*HSP70 (raised in mouse; [5], detecting the parasite cytoplasmic heat-shock protein 70 and anti-*Pb*MSP-1 (raised in mouse; MRA-667 from MR4; www.MR4.org), detecting the merozoite surface protein 1 of *P. berghei*. The anti-UIS-4 antibody were preferred over the earlier described anti-EXP-1 antibody [9], detecting another PVM resident protein because of the intensity and the constitutive expression. Anti-mouse and anti-rabbit secondary antibodies, conjugated to Alexa-488 and Alexa-594, were used for visualization (Invitrogen). Nuclei were stained with DAPI. Analysis of infected hepatocytes was performed using a Zeiss Axiophot Fluorescence microscope with Axiocam MRm CCD (Fig. 1C and Fig S1) camera or a Olympus FV1000 Confocal Laser Scanning Microscope.

### TEM Analysis of Infected Huh-7 Cells

For ultrathin-section transmission electron microscopy, 2×10^5^ wt and 5×10^5^ *p52/p36*-deficient sporozoites were used to infect 3.5×10^5^ sub-confluent Huh-7 cells, seeded the day prior in 35 mm petridishes. Sporozoites were centrifuged for 10 minutes at 1800×G and 32 hours post infection cells were fixed in 2.5% glutaraldehyde (Electron Microscopy Sciences) in 0.1 M sodium cacodylate buffer (pH 7.4) for 1 h at room temperature and subsequently washed three times for 10 minutes in 0.1 M sodium cacodylate buffer and then post-fixed for 1 h in 1% osmium tetroxide (Electron Microscopy Sciences, Gibbstown, NY) in sodium cacodylate buffer at room temperature. Samples were washed three times 20 minutes in 0.1 M sodium cacodylate buffer and subsequently dehydrated in a graded series (10-50-70-96-100%) of ethanol. Cells were resin infiltrated in a 100% ethanol/EPON (Sigma) mixture (2∶1) for 3 hours and subsequently in a 100% ethanol/EPON mixture (1∶1) for 5 hours and subsequently in pure EPON overnight. Beem capsules were placed onto the cells perpendicular, filled with EPON, and polymerized overnight at 60°C. Ultrathin (50–100 nm) sections were cut parallel to the cell surface using an Ultracut ultramicrotome (Leica, Germany) and contrasted with 2% uranyl acetate and lead citrate before examination with a JEOL 1010 microscope under 60 kV.

### Analysis of Infectivity of Huh-7 Hepatoyte-derived Merozoites

Assessment of the infectivity of hepatocyte derived merozoites has previously been described for *PbΔlisp1* mutants [12]. The protocol was adapted and Huh-7 cells were seeded in a 24-wells plate at 10^6^ cells/well, overnight. Sporozoites were added to the wells (>80% confluent) at 8×10^4^ sporozoites per well, and centrifuged 10 minutes at 1800×G (eppendorf centrifuge 5810 R). 65 hours post infection 100 µl supernatants were collected from each well, centrifuged for 3 minutes at 12.000 rpm and the cell pellet was re-suspended in 100 µl RPMI. A total of 200 µl re-suspended culture supernatant (from 2 wells) was injected i.v per C57BL/6 mice. Approval was obtained from the Radboud University Experimental Animal Ethical Committee (RUDEC 2009-225). Blood stage infections were monitored by Giemsa staining of blood smears from day 2 up to day 14 post injection. Genotype confirmation of *Δp*52&p36 and wildtype parasites was performed as described [9]. The pre-patent period was defined as the period of time (days) between injection and the day that mice showed a blood stage parasitemia of 0.5–2%.

## Results

### *P. berghei* Δp</emph>52&p36* Parasites can Partially Develop Inside the Nucleus of the Hepatocyte*

*In vitro* analysis of *P. berghei* infected Huh-7 hepatocyte cultures showed that compared to wildtype (100%), a low proportion of Δ*p*52&p36 sporozoites, (2±0.6% (p<0.01)) was able to develop into replicating intra-hepatic parasites (Fig. 1a, Table S1). Most knockout parasites (98%) abort development soon after invasion and do not start nuclear replication.

Remarkably, a relatively large proportion of the replicating Δ*p*52&p36 parasites, 45% (±0.7%) resided inside the nucleus of hepatocytes, compared to 1.25% (±0.35%) of intranuclear wildtype parasites (p<0.01) at 24 hours post invasion (Fig. 1a, Table S1). The absolute number of intranuclear mutant parasites matched the number of wildtype parasites. For both wildtype and mutant parasites, there was a slight decrease in the percentage of intranuclear developing parasites during the course of parasite maturation. At any time point, however, while the absolute number remained the same, the percentage of intranuclear mutant parasites was significantly higher than the percentage of intranuclear wildtype parasites (p<0.05) (Fig. 1a).

Intranuclear developing *P. berghei* wildtype and Δ*p*52&p36 parasites were negative for UIS-4 peripheral staining, a marker for the presence of a PVM (Fig. 1b) and did not express MSP-1 at 52 hours post infection, as depicted by an intranuclear *Δp*52&p36 parasite (Fig. 1c). At time points up to 72 hours post infection, these parasites remained negative (data not shown) indicating that the absence of MSP1 staining is not the results of a delay in maturation period. Based on MSP-1 expression, intranuclear parasites are unlikely the cause of Δ*p*52&p36 parasite breakthrough in mice.

### Cytosolic Δp</emph>52&p36* Parasites can Produce Mature Merozoites in the Absence of an Apparent PVM*

**Figure 2:**
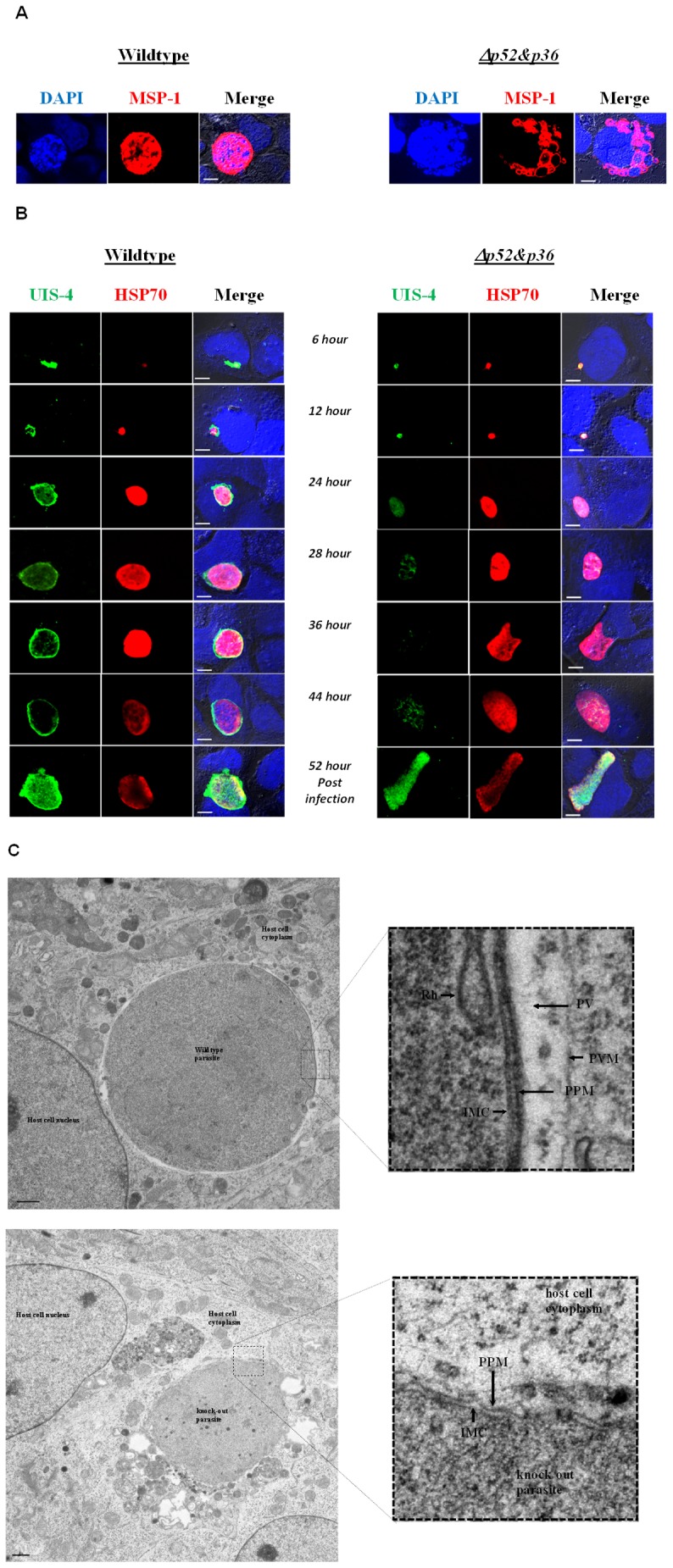
Cytosolic developing *Δp52&p36 P. berghei* parasites lack an apparent PVM. A) MSP-1 expression on cytosolic wildtype and *Δp52&p36 P. berghei* parasites 52 hours post infection (Bar  = 10 µm). B) UIS-4 and HSP70 expression on cytosolic *Δp52&p36* and wildtype *P. berghei* parasites at 6–52 hours post infection (Bar  = 10 µm). C) Electron microscopic analysis of cytosolic wildtype (upper row) and *Δp52&p36* (lower row) parasites, 32 hours post hepatocyte infection. The inset boxes show higher magnifications of the boxed areas within the overview images. IMC, inner membrane complex; Ly lysosome; NE, Nuclear envelope; PPM, parasite plasma membrane; PV, parasitophorous vacuole; PVM, parasitophorous vacuole membrane; Rh, rhoptry (Bar  = 10 µm).

More than half of the replicating Δ*p*52&p36 parasites resided in the cytosol of Huh-7 hepatocytes (Fig. 1a) expressing MSP-1 and transforming into mature merozoites from 52 hours post invasion onwards. These cytosolic Δ*p*52&p36 parasites did not show the typical round shape of wildtype parasites, but were instead characterized by an irregular morphology (Fig. 2a, Fig S1). Individual merosomes were clearly visible budding of from the infected hepatocyte (Fig S1 right box). Replicating cytosolic Δ*p*52&p36 parasites (n = 498) were negative for peripheral UIS-4 staining at any time point starting from early liver infection onwards (6–52 hour post invasion) (Fig. 2b). Using transmission electron microscopy at 32 hours post infection, we observed cytosolic wildtype parasites demarked by a surrounding PV and PVM, while, in contrast, both PV and PVM could not be detected in Δ*p*52&p36 parasites (Fig. 2c). Thus, Δ*p*52&p36 parasites replicating in the cytosol expressed MSP-1, but lacked an apparent PVM.

### Hepatocyte Derived Δp</emph>52&p36* Merozoites are able to Induce a Blood Stage Infection*

We next tested whether Δ*p*52&p36 parasites developing into merozoites were capable of infecting erythrocytes. Therefore, supernatants of Δ*p*52&p36 and wildtype infected Huh-7 cells, collected 65 hours post infection, were injected i.v in C57BL/6 mice (Table 1). All mice injected with culture supernatant became patent with blood stage parasitemia as determined by thick smear. Genotyping of blood parasites confirmed the Δ*p*52&p36 genotype (Fig. S2). The mean difference in day of patency between Δ*p*52&p36 and wildtype parasites i.e. 5.9 versus 2.4 days post injection respectively, likely reflects the difference in number of viable merozoites injected. These data show that Δ*p*52&p36 parasites, developing in Huh-7 hepatocytes in the absence of an apparent PVM, are capable of maturing into infectious merozoites.

**Table 1:** *Δp52&p36* merozoites are capable of inducing a blood-stage infection.

Experiment no.	No. Asexual positive/No.injected (mean±sd pre-patency)	
	***Δp52&p36***	**WT**
**1**	4/4 (6±0 days)	2/2 (3±0 days)
**2**	5/5 (5.8±0.4 days)	3/3 (2±0 days)

Huh-7 cells were infected with *Δp52&p36* and WT parasites and cultured for 65 hours. After 65 hours, culture supernatant containing merozoites was collected and injected i.v in C57BL/6 mice. Regular Giemsa staining was performed in all groups, 2–14 days post i.v injection in mice, to control for asexual parasites.

## Discussion

Here we show that a proportion of *P. berghei* Δ*p*52&p36 parasites can develop in Huh-7 hepatocytes in the apparent absence of a PVM and fully mature into merozoites. Merozoites derived from an *in vitr*o Δ*p*52&p36 hepatocyte culture were infectious and lead to a blood stage infection in mice. Our data question the absolute necessity for the presence of a PVM for intrahepatic *P. berghei* development.

Although all observed replicating Δ*p*52&p36 parasites herein (approximately 900 by immunofluorescence) develop free of PVM inside the hepatocytes, one cannot formally exclude the possibility that a small proportion of the *P. berghei* mutants develop with a PVM into infectious merozoites. Nonetheless, the breakthrough blood stage parasites were infectious for mosquitoes and upon re-infection of a hepatocyte culture with sporozoites, all replicating mutants lacked UIS-4 staining (data not shown). This indicates that the infectious merozoites did not arise from a subset of mutant parasites that stably switched to a phenotype of forming a PVM in the absence of P52 or P36.

The relatively high percentage of mutant parasites replicating in the hepatocyte nucleus seems remarkable. However, despite an apparent preference of the mutant parasites to replicate in the nucleus, the absolute number of intranuclear replicating mutants does not differ from wildtype. Thus, mutant parasites do not preferentially invade the nucleus of hepatocytes.

The seemingly preference for intranuclear development merely arises from the developmental arrest of a major part of the cytosolic mutant parasites. Intranuclear mutants have a developmental advantage over cytosolic mutant parasites and their numbers seem untouched by the absence of a PVM. Possibly, the nuclear envelope acts as a substitute for the PVM.

The intranuclear mutant and wildtype *P. berghei* parasites likely halt inside the nucleus of the hepatocyte upon transmigration, similar to *P. yoelii* and *P. falciparum* [4]. These parasites do probably not play a role in the observed breakthrough infections in mice. Based on MSP-1 expression, cytosolic and not intranuclear parasites are the likely source of infectious merozoites. Nevertheless, we cannot exclude that merozoites may arise from intranuclear developing parasites.

Whereas *Plasmodium* parasites replicating in the nucleus in the apparent absence of a PVM have previously been reported, cytosolic parasites have not. Interestingly, upon close examination of wildtype parasites, a small percentage (±1.5% n = 27) of the cytosolic wildtype parasites lack peripheral UIS-4 staining at 30 hour post infection, similar to the cytosolic Δ*p*52&p36 parasites (data not shown). These data support the possibility of a non-conventional intra-hepatic pathway for *P. berghei* development.

In the absence of an encompassing membrane cytosolic parasites are likely more vulnerable to host defense mechanisms. Genes involved in the evasion of host cell apoptosis [13], [14] might be less effective to avert cell death. Possibly, replicating mutant parasites survive in an equilibrium of anti-apoptotic gene expression and host defense mechanism.

It remains to be seen whether these findings in *P. berghei* are representative for other *Plasmodium* species. Fully developing cytosolic parasites absent of a PVM have, to our knowledge, never been reported for any of the other *Plasmodium* species. While *P. yoelii* Δ*p*52&p36 breakthrough has not been reported, Labaied *et al.* previously described a multi-nucleated *P. yoelii* ‘growth arrested’ Δ*p*52&p36 parasite in a Hepg2-CD81 cell [8], a human hepatoma cell line that promotes the formation of a PVM by *P. yoelii* [4]. This particular parasite lacked a peripheral UIS-4 expression [8]. Apparently, very low numbers of the mutant *P. yoelii* parasites still replicate inside Hepg2-CD81 cells in the absence of a PVM. Furthermore, it will be interesting to see whether the low numbers of mutant Δ*p*52&p36* P. falciparum* parasites that replicate in primary human hepatocytes [9] have a similar phenotype as the *P. berghei* mutants.

Our findings are confined to an *in vitro* Huh-7 *P. berghei* model and the *in vivo* relevance of these findings remains elusive. *In vivo* characterization is hampered by the extremely low number of replicating parasites. Similarly, *P. yoelii* and *P. falciparum* replicating Δ*p*52&p36 parasites are extremely rare and at present their mechanism of breakthrough remains unclear. Nevertheless, once confirmed in *P. falciparum*, our findings may have implications for the development of a genetically attenuated malaria vaccine. Based on protective efficacy conferred in mice and apparent full arrest in *P. yoelii* and *P. falciparum* models, genetically attenuated *Δp*52&p36 parasites have been considered eligible for clinical development as an attenuated sporozoite vaccine [7]. Given the break-through infections, our data suggest that for a sufficiently attenuated malaria vaccine, multiple genes need to be targeted. Such genes could not only include genes involved in the formation of the PVM, but preferably other *Plasmodium* gene targets with independent functions for liver stage development.

## Supporting Information

Figure S1 **Late liver stage intracytosolar** ***Δp***52&p36*p* **parasites have an irregular shape.** Four representative images of *Δp*52&p36*p P. berghei* parasites in culture 48 hours post invasion in Huh-7 cells. Msp-1 expression is depicted in red, DAPI in blue (Bar = 10 µm).
(TIF)

Figure S2 **Confirmation of Δ** ***p52+p36*** **and wildtype genotype after merosome injection assay.** A) Diagnostic PCR for confirmation of correct disruption of *p52* and *p36* in mutant Δ*p52+p36* (1409cl1). SM: selectable marker (primers 4501/4502; 1093bp); 5′-integration event (primers L1389/L313; 1050bp); ORF (primers L775/L121; 1029bp). B) Sequence of the primers used. C) Southern analysis of pulse field gel (PFG)-separated chromosomes of mutant Δ*p52+p36*. Mutant Δ*p52*+*p36* has been generated in the reference *P. berghei* ANKA line PbGFP-Luccon which has a *gfp-luciferase* gene integrated into the silent 230p locus (PBANKA_030600) on chromosome 3 (*i.e.* RMgm-29; http://pberghei.eu/index.php?rmgm=29). Hybridization with the 3′-UTR *dhfr/ts* probe recognizes the integrated construct on chromosome 9, the reporter GFP-Luc_con_ construct on chromosome 3, and the endogenous *dhfr/ts* gene located on chromosome 7.
(TIF)

Table S1 Quantitative analysis of replicating intranuclear and cytosolic wildtype and mutant parasites. ^a^ Average number of replicating liver stage parasites per coverslip. A total of 3 coverslips was counted per timepoint per parasite.
(DOC)
