# Supplementary figures and images for: Plasmodium berghei Δp52&p36 Parasites Develop Independent of a Parasitophorous Vacuole Membrane in Huh-7 Liver Cells
Source: PLoS One. 2012 Dec 5;7(12):e50772. doi: 10.1371/journal.pone.0050772 (PMC3515443; doi:10.1371/journal.pone.0050772)

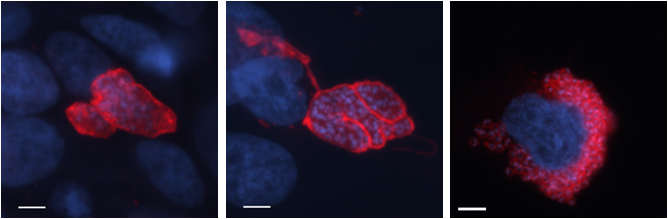

Supplement: Figure S1 — Late liver stage intracytosolar Δp52&p36p parasites have an irregular shape. Four representative images of Δp52&p36p P. berghei parasites in culture 48 hours post invasion in Huh-7 cells. Msp-1 expression is depicted in red, DAPI in blue (Bar = 10 µm). (TIF) [file pone.0050772.s001.tif]

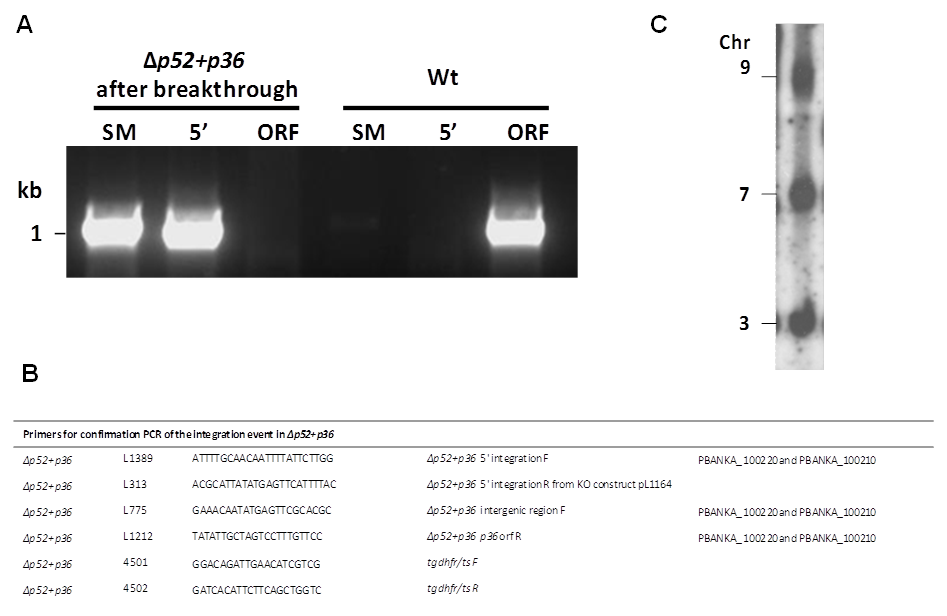

Supplement: Figure S2 — Confirmation of Δ p52+p36 and wildtype genotype after merosome injection assay. A) Diagnostic PCR for confirmation of correct disruption of p52 and p36 in mutant Δp52+p36 (1409cl1). SM: selectable marker (primers 4501/4502; 1093bp); 5′-integration event (primers L1389/L313; 1050bp); ORF (primers L775/L121; 1029bp). B) Sequence of the primers used. C) Southern analysis of pulse field gel (PFG)-separated chromosomes of mutant Δp52+p36. Mutant Δp52+p36 has been generated in the reference P. berghei ANKA line PbGFP-Luccon which has a gfp-luciferase gene integrated into the silent 230p locus (PBANKA_030600) on chromosome 3 (i.e. RMgm-29; http://pberghei.eu/index.php?rmgm=29). Hybridization with the 3′-UTR dhfr/ts probe recognizes the integrated construct on chromosome 9, the reporter GFP-Luccon construct on chromosome 3, and the endogenous dhfr/ts gene located on chromosome 7. (TIF) [file pone.0050772.s002.tif]
