# Supplementary material for: Plasmodium berghei Δp52&p36 Parasites Develop Independent of a Parasitophorous Vacuole Membrane in Huh-7 Liver Cells
Source: PLoS One. 2012 Dec 5;7(12):e50772. doi: 10.1371/journal.pone.0050772 (PMC3515443; doi:10.1371/journal.pone.0050772)
Supplement: Table S1 — Quantitative analysis of replicating intranuclear and cytosolic wildtype and mutant parasites. a Average number of replicating liver stage parasites per coverslip. A total of 3 coverslips was counted per timepoint per parasite. (DOC) [file pone.0050772.s003.doc]

| **Parasite** | **Wildtype** | | **Mutant** | | |
| --- | --- | --- | --- | --- | --- |
| **24 hr post infection** | **Total No. Parasites**  **+/- SD ^a^** | | **Total No. Parasites**  **+/- SD ^a^** | | |
|  | 1420  +/- 82 | | 27.5  +/- 8 | | |
|  | **% Intranuclear**  **(±SD)** | **% Cytosolic**  **(±SD)** | **% Intranuclear**  **(±SD)** | | **% Cytosolic**  **(±SD)** |
|  | 1.25%  (±0.35%) | 98.75%  (±0.35%) | 45.5% (±2.7%) | | 54.5%  (±2.7%) |
| **36 hr post infection** | **Total No. Parasites**  **+/- SD ^a^** | | **Total No. Parasites**  **+/- SD ^a^** | | |
|  | 1099  +/- 58 | | 23  +/- 2 | | |
|  | **% Intranuclear**  **(±SD)** | **% Cytosolic**  **(±SD)** | **% Intranuclear**  **(±SD)** | **% Cytosolic**  **(±SD)** | |
|  | 0.75%  (±0.07%) | 99.25%  (±0.07%) | 31.5%  (±6.4%) | 68.5%  (±6.4%) | |
| **48 hr post infection** | **Total No. Parasites**  **+/- SD ^a^** | | **Total No. Parasites**  **+/- SD ^a^** | | |
|  | 898  +/- 53 | | 21  +/- 5 | | |
|  | **% Intranuclear**  **(±SD)** | **% Cytosolic**  **(±SD)** | **% Intranuclear**  **(±SD)** | **% Cytosolic**  **(±SD)** | |
|  | 0.15%  (±0.07%) | 99.85  (±0.07%) | 29%  (±4.2%) | 71%  (±4.2%) | |
